# Crystalloid podocytopathy with focal segmental glomerulosclerosis in PCM: a case report

**DOI:** 10.1186/s13000-015-0448-0

**Published:** 2015-12-16

**Authors:** You La Jeon, Woo In Lee, Yujin Choi, So Young Kang, Myeong Hee Kim, Sung-Jig Lim, Sang Ho Lee

**Affiliations:** Department of Laboratory Medicine, School of Medicine, Kyung Hee University and Kyung Hee University Hospital at Gangdong, 892, Dongnam-ro, Gangdong-gu, Seoul, 134-727 Republic of Korea; Department of Pathology, School of Medicine, Kyung Hee University and Kyung Hee University Hospital at Gangdong, Seoul, Republic of Korea; Division of Nephrology, Department of Internal Medicine, School of Medicine, Kyung Hee University and Kyung Hee University Hospital at Gangdong, Seoul, Republic of Korea

**Keywords:** Plasma cell myeloma, Crystalline deposition, Focal and segmental glomerular sclerosis, Podocytopathy, Tubulopathy, Bone marrow

## Abstract

**Background:**

Crystalloid podocytopathy with focal segmental glomerulosclerosis in plasma cell myeloma (PCM) is rare.

**Case Presentation:**

We present a case of crystalline deposition in the bone marrow (BM) and various renal cells with only proteinuria as a symptom. As workup for proteinuria, a renal biopsy sample was obtained. EM showed multiple crystalline depositions in renal tubular cells and podocytes. Focal segmental glomerulosclerosis with crystalloid podocytopathy was diagnosed. Because monoclonal gammopathy was detected in the serum and urine, a BM study was also performed. Plasma cells with needle-shaped inclusion bodies were observed. The crystalline deposits in the plasma cells and podocytes were positive for Masson’s trichrome and kappa light-chain staining. These findings indicated that the crystalline deposits originated from paraprotein. The case showed a rare process of focal segmental glomerulosclerosis via crystalline deposition in podocytes in plasma cell myeloma.

**Conclusions:**

Crystalloid podocytopathy is a likely cause of renal damage such as FSGS in PCM, although it is an uncommon mechanism for myeloma kidney.

## Background

Monoclonal proteins may take various shapes and forms, such as immunoglobulin (Ig) aggregates, amyloid substances, or even crystalline deposits. In particular, in the rare cases, crystalline deposition can be found in plasma cell myeloma (PCM) as initial presentation of the disease or as a complication in the bone marrow (BM), kidney, or other organs [1]. Occasionally, intracellular crystalline deposition has been found in myeloma cells and histiocytes among BM hematopoietic cells [2]. The kidneys are more rarely affected with macrophages, glomerular cells, or proximal tubular cells [3, 4]. Furthermore, reports of cases involving crystalline deposition in podocytes (or glomerular visceral epithelial cells) are extremely rare [5]. Here, we report a rare case of focal segmental glomerulosclerosis (FSGS) wherein multiple crystalline inclusions were seen in BM plasma cells and tubular epithelial cells and podocytes in the kidney in a patient with PCM.

## Case presentation

A 52-year-old woman was referred to a nephrologist for work-up of proteinuria and slightly increased serum creatinine levels found during a routine health examination. The patient had been aware of her proteinuria for 2 years. However, she had not undergone further evaluation or treatment. The patient had no other relevant medical history.

The laboratory results of complete blood count and blood chemistry assay were represented in Table 1. Urinalysis revealed 1+ protein with normal pH, and the results were negative for glucose. Random urine chemistry showed a state of glomerular proteinuria as the results revealed creatinine level of 1847.56 μmol/L (20.9 mg/dL), microalbumin level of 0.04869 g/dL (486.9 mg/L), and an albumin/creatinine ratio of 2329.7 mg/g. The protein level in the 24-h urine sample was elevated to 2.62 g. The patient was then found to have a monoclonal protein concentration of 5.2 g/L in serum and 0.01 g/L in urine. The serum and urine monoclonal protein fractions were found to represent IgG and kappa in immune fixation electrophoresis. The free kappa and lambda light-chain levels in the serum were 571.6 mg/L and 23.3 mg/L, respectively, and the serum free light-chain ratio was 24.53 (0.26 − 1.65). There was no evidence suggesting hepatitis B or C virus or human immunodeficiency virus infection on serologic tests.

**Table 1:** The laboratory results of complete blood count and blood chemistry

The test	Results	Reference range
Complete blood count		
WBC count	4.2 × 10^9^ /L	4.0 - 10.0
Hemoglobin	111 g/L	120 - 160
Platelet count	180 × 10^9^ /L	350 - 350
Blood chemistry		
Protein	63 g/L	66 - 83
Albumin	33 g/L	35 - 52
Creatinine	115.8 μmol/L	44.2 - 79.6
Calcium	2.2 mmol/L	2.2 - 2.7
Phosphorus	1.1 mmol/L	0.8 - 1.5
Sodium	136 mmol/L	136 - 146
Potassium	3.8 mmol/L	3.5 - 5.1
Chloride	108 mmol/L	101 - 109
Uric acid	208.2 μmol/L	154.7 - 356.9
BUN	5.4 mmol/L	2.86 - 7.14
Ig G	12.2 g/L	0.7 - 1.6
Ig A	870 mg/L	700 - 4000
IgM	640 mg/L	400 – 2300

*Abbreviations*: *WBC* white blood cell, *BUN* blood urea nitrogen, *Ig* immunoglobulin

**Fig. 1:**
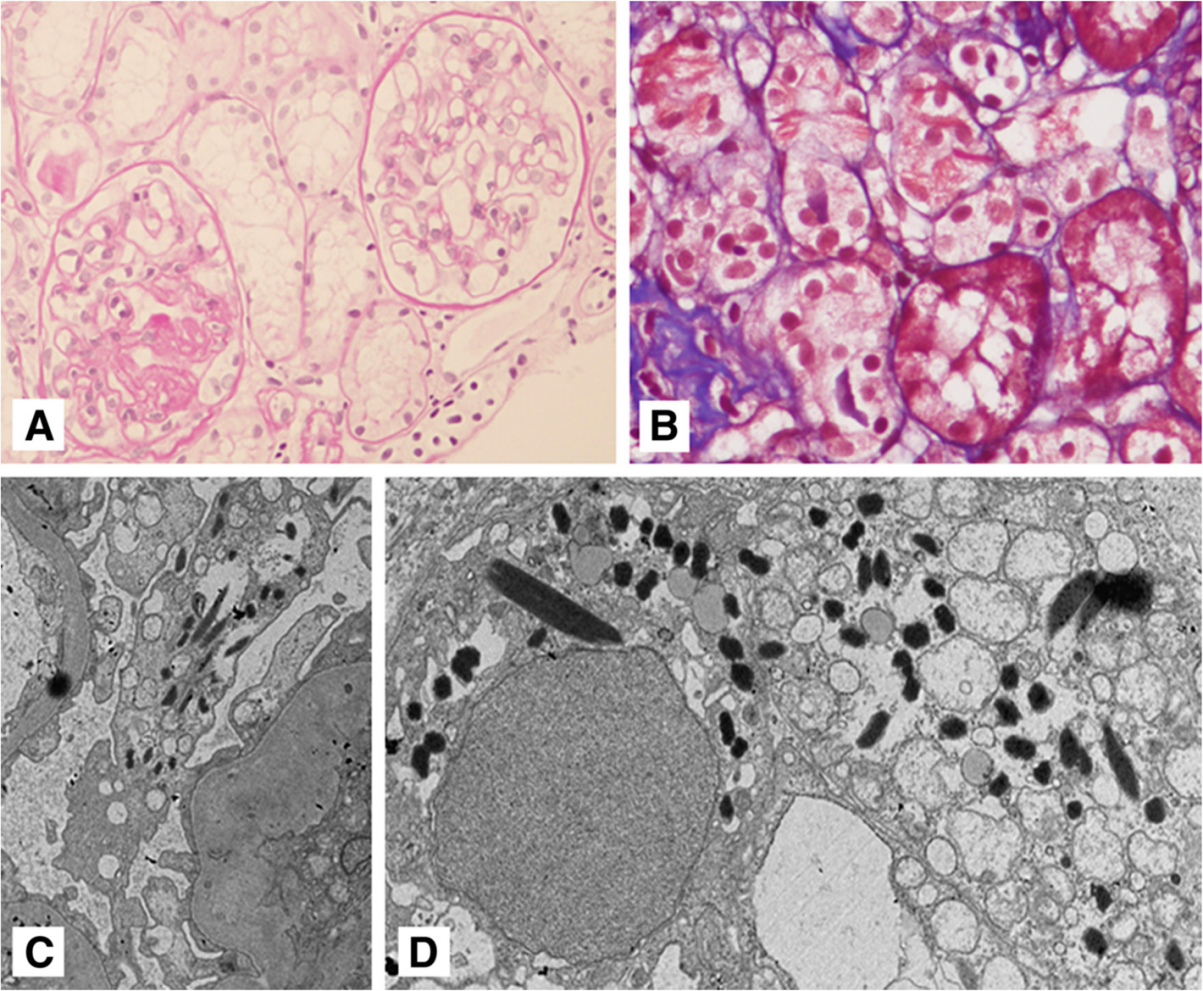
**a** Some glomeruli show segmental or global glomerulosclerosis (periodic acid–Schiff stain, ×400 magnification). **b** Fine needle-shaped crystalline structures in proximal tubular epithelial cytoplasm, which were negative for periodic acid-Schiff staining (Masson’s trichrome stain, ×400). Crystalline deposition was observed in podocytes (**c**) and proximal tubular epithelial cells with many large and abnormal lysosomes (**d**). The morphology of the crystalline structures was diverse and ranged from a needle shape to a rhomboid shape (electron micrograph, original magnification: **c**, ×5,000; **d**, ×6,000)

**Fig. 2:**
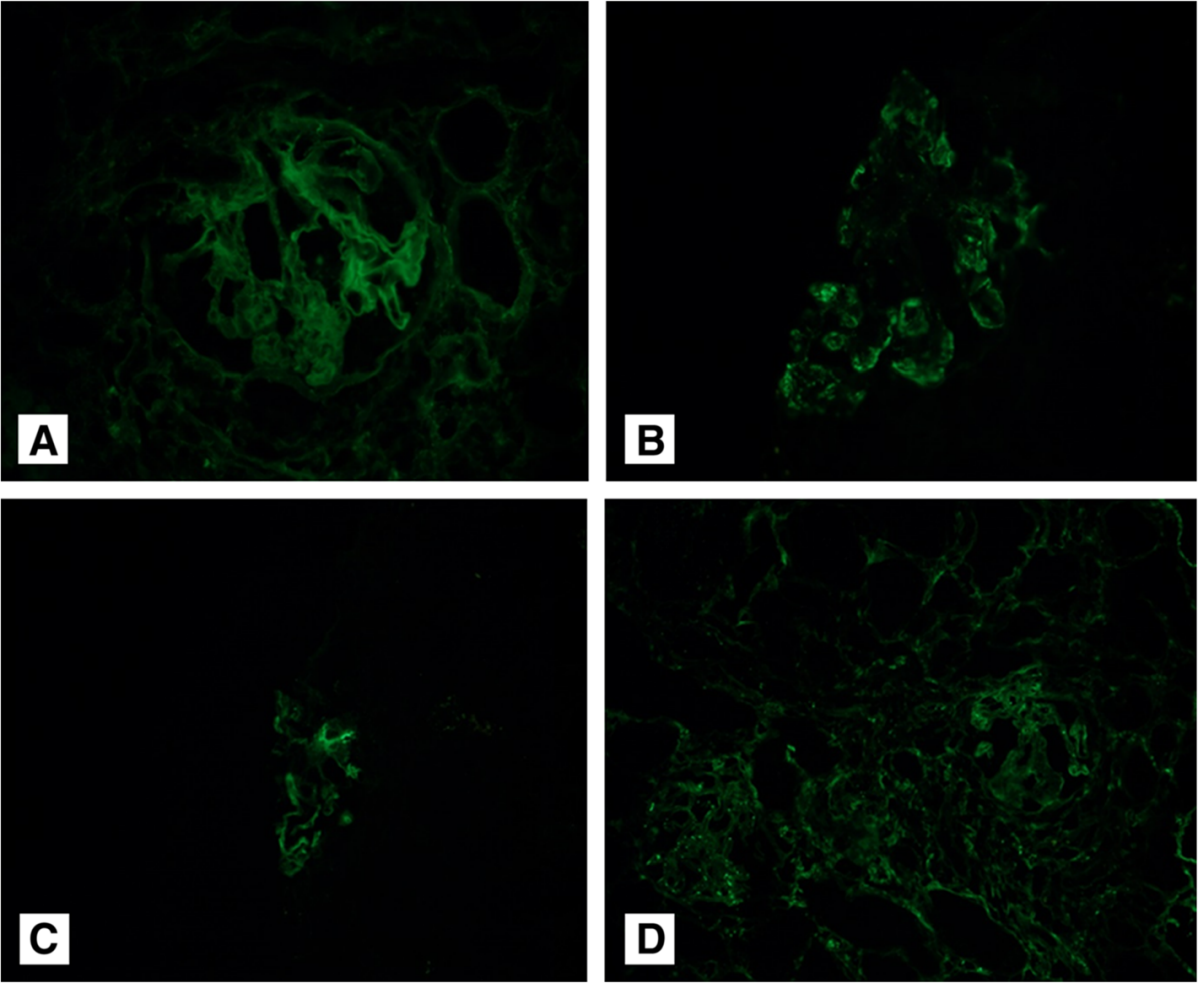
Immunofluorescence microscopy of the glomerulus. **a** A linear pattern for IgG (trace) (x200 magnification). **b**, **c**, and **d**, A granular pattern for complement component C3 (++), C1q (+), and for fibrinogen (trace) (**b**, x200; **c**, x100; **d**, x100 magnification)

An ultrasound-guided kidney biopsy was performed; the final pathologic diagnosis was FSGS. Among 16 glomeruli, seven showed global sclerosis and two showed segmental glomerulosclerosis (Fig. 1a). Mild tubular atrophy, mild interstitial fibrosis, and focal tubular necrosis were observed. Interestingly, proximal tubular epithelial cells contained some crystalline structures (Fig. 1b). Congo red staining also produced negative results. Interstitial inflammation with mild infiltration of lymphocytes was noted. Immunofluorescence microscopy showed a linear pattern with trace intensity for IgG and a granular pattern for complement component C3 (++), C1q (+), and fibrinogen (trace) in the glomerulus (Fig. 2). However, there was no significant positive staining for IgA, IgM, or C4. On performing immunofluorescent staining, the mesangium, tubules, interstitium, and vessels were negative for staining. However, many of the tubular cells showed stronger positivity for the kappa light chain than for the lambda light chain on immunohistochemical staining.

Electron microscopic examination revealed diffuse effacement of foot processes and electron-dense deposits, with some crystalline structures in the cytoplasm of visceral glomerular epithelial cells. Furthermore, similar electron-dense crystalline deposits found in podocytes were observed throughout the cytoplasm of proximal tubular cells (Fig. 1c and d). These showed diverse morphology with a needle shape or a rhomboid shape and variable size ranging from 0.2 × 1 to 0.5 × 2.8 μm^2^. However, no pathologic abnormalities were noted within the glomerular basement membrane, mesangium, or glomerular endothelium.

**Fig. 3:**
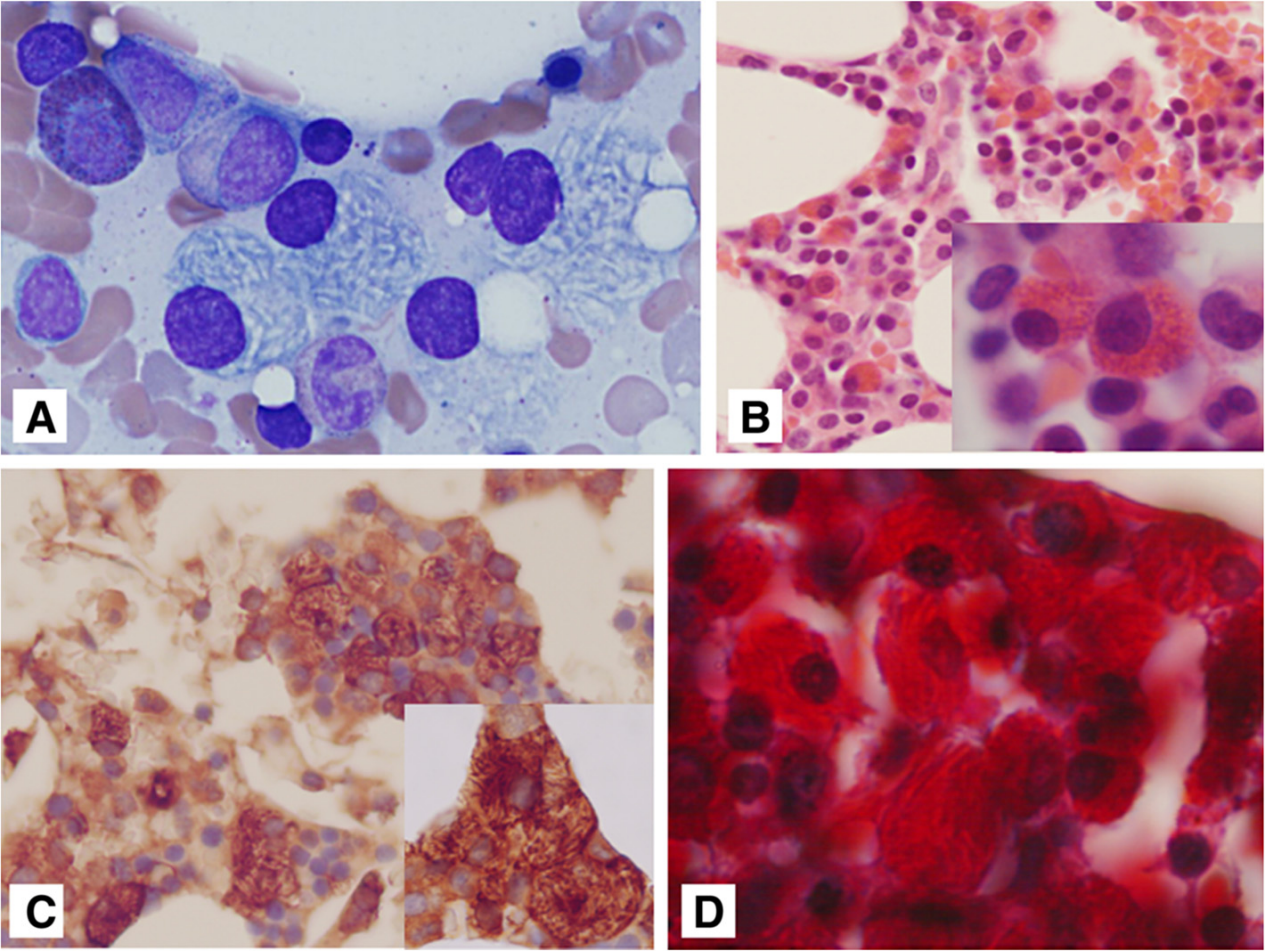
**a** Plasma cells with fine needle-shaped intracellular crystalline depositions were frequently seen in the bone marrow (Wright’s stain, ×1,000 magnification). **b** The red-colored cytoplasmic inclusions and eccentric mononucleosis were frequently observed in the clot sections (hematoxylin and eosin staining, (**b)** × 400 magnification) (inset, ×1,000 magnification). **c** The plasma cells were stained strongly for the kappa light-chain. Some fine needle-shaped crystalline depositions were also observed in the cells (×400 magnification) (inset, ×1,000 magnification). **d** The deep-red-colored cytoplasmic inclusions with fine needle-shaped structures considered to be crystalline deposits (Masson's trichrome stain, ×1000)

A BM examination was performed to investigate for the presence of plasma-cell neoplasms. The BM aspirate smears were stained with Wright’s stain. The plasma cells with fine needle-shaped intracellular crystalline depositions were found to have increased in number, representing up to 10.4 % of the total nucleated cells counted (Fig. 3a). Typical plasma cells were rarely observed. All of the cells with intracellular cytoplasmic crystalline inclusions were negative for α-naphthyl acetate esterase (ANBE) staining, suggesting that macrophages such as histiocytes were not involved. The paraffin-embedded BM biopsy and clot sections were stained with hematoxylin and eosin. The cellularity of the BM was 30–40 %, that is, she had normocellular marrow for her age. Cells with red-colored cytoplasmic inclusions and eccentric mononucleosis, which seemed to be malignant plasma cells, were frequently seen in the clot sections (Fig. 3b). Immunohistochemical staining was performed on the BM clot sections with antibodies against CD138, kappa light-chain, and lambda light-chain. Plasma cells stained with CD138 were seen in these sections more frequently than in the aspiration smear. The plasma cells showed strongly positive staining for kappa light-chains (Fig. 3c). However, a few of the plasma cells were positive for lambda light-chain staining. Masson’s trichrome staining was performed on clot sections to evaluate the characteristic features of the crystalline deposits. The plasma cells had deep-red-colored cytoplasmic inclusions with fine needle-shaped structures (Fig. 3d). These cells were negative in periodic acid-Schiff staining.

The complex karyotype of 44,X,-X,-6,-21,+mar[2]/46,XX[53] was determined by conventional karyotyping using the BM specimen. The fluorescence *in situ* hybridization studies for detecting cytogenetic abnormalities such as *TP53* deletion, 13q14 (*RB1*) deletion, 1q trisomy, and rearrangement of *IgH/MAF* - t(14;16), *IgH/FGFR3* - t(4;14), *IGH/CCND1* - t(11;14) were found to be negative.

## Discussion

A portion of patients with renal disease are diagnosed with PCM [6]. Approximately 60 % of PCM patients have renal failure, and proteinuria can be found in 90 % of these patients [7]. Most of the cases of PCM with renal involvement show myeloma cast nephropathy (light-chain cast nephropathy) and low-molecular-weight proteinuria [8]. Amyloidosis and monoclonal Ig deposition disease are seen less commonly. Moreover, crystalline deposition is an even more rare presentation. In previous reports, the organs shown to be involved in PCM were the kidneys, skin, liver, lung, spleen, and stomach and various other tissues such as those of the cornea. [9-11]

Except tubular luminal involvement (myeloma cast nephropathy), the main pathologic feature accompanying crystalline deposition in the kidney is light-chain-associated Fanconi syndrome [12]. Light chains secreted by monoclonal plasma cells are reabsorbed by proximal tubular epithelial cells, and on rare occasions, intracellular crystals are formed during this process. These crystals impair tubular function and disrupt overall reabsorption capabilities causing various aggressive symptoms such as glycosuria, aminoaciduria, hyperuricosuria, hyperphosphaturia, and hypercalciuria [13]. In most of these patients, kappa light-chains are typically involved as components of their monoclonal proteins [14, 15]. The variability subtype Vk1 has the property of resistance to proteolysis by the lysosomal enzyme cathepsin B in tubular epithelial cells [15]. This characteristic of Vk1 is due to germline mutations of *O2/O12* or *O8/O18* [14]. Among these genes, a major mutation of *O2/O12* induces a nonpolar or hydrophobic residue at position 30, which represents complementarity-determining region L1 of light chains and forms a portion of the Ig antigen-binding site [13, 14].

Interestingly, our patient showed crystalline deposition in podocytes as well as proximal tubular epithelial cells. Crystalloid podocytopathy is rare, but may occur due to its similar endocytic function of the proximal tubular epithelial cells [16]. Recent studies have reported that in healthy rats, an amount of albumin passes through the glomerular filtration barrier [17]. Podocytes take up albumin and other plasma proteins and process these through degradation or transcytosis [16]. The degradation of albumin occurs in the lysosomes of podocytes [18]. Monoclonal proteins are also considered to be processed in the same manner. If any of these monoclonal proteins are resistant to proteolysis, this may result in crystal formation. Furthermore, the membranous endocytic receptors of proximal tubular epithelial cells, such as megalin, cubilin, and CIC-5, have been found in human podocytes [19].

Podocytopathy caused by crystalline deposits is a very rare phenomenon and only ten cases have been reported in the literature so far [4, 5, 9-11, 20-24]. The reported cases of crystalloid podocytopathy always involved additional crystalline deposition in other cells in the kidney. Accompanying foot process effacement was common (eight cases out of 11, including the present case), and sclerotic glomeruli implying FSGS was also a common finding (eight cases out of 11). FSGS was considered to occur by crystalloid podocytopathy and paraproteinemia itself in our patient because no other causative factors such as viral infection, drug therapy, or toxins were involved [25]. Proteinuria of various degrees was noted, and over 24 h, the urine protein levels ranged from 0.31 mg to 14.4 g. In this case, the patient presented with glomerular proteinuria, which could be attributed to the crystalline deposition of podocytes.

Although the majority of the cases of podocytopathy caused by crystalline deposits had concurrent tubulopathy (six cases out of 11 had obvious signs of tubulopathy, three cases had no definite description of proximal tubule injury, one case had distal tubulopathy, and one case had no tubulopathy), there was only one case of Fanconi syndrome. Our patient showed only proteinuria with renal insufficiency. It is possible for distributed accumulation of pathologic light-chains in both the glomeruli and tubules to have caused these findings. Such conditions may induce milder impairment of proximal tubular function than sole presentation of proximal tubulopathy.

A morphologically distinguishable feature of this case was the crystalline deposits in BM plasma cells. Among previous cases with crystalloid podocytopathy, only four were described with such findings. Plasma cells may possess crystallized cytoplasmic Ig in their abundant endoplasmic reticulum with various shapes of crystal [2]. The presence of crystalline deposits does not definitively imply the existence of malignant plasma cell dyscrasia because they can also be observed in reactive plasma cells [26]. In fact, the presence of these deposits could be a favorable prognostic finding, along with slow and prolonged disease course, due to the accompaniment of decreased secretion of monoclonal proteins [27]. However, the proportion of patients who had plasma cells with cytoplasmic crystalline deposition in crystalloid podocytopathy (four cases out of 11) or tubulopathy (13 cases out of 57) in a previous study was not low, and the exact duration of these diseases is mostly unknown [14].

## Conclusion

Our patient’s sole problem was a proteinuria, besides glomerular proteinuria. Multi-directional clinical, laboratory, and pathologic diagnostic methods contribute to making an accurate diagnosis and understanding pathophysiology. Crystalloid podocytopathy is a likely cause of renal damage such as FSGS in PCM, although it is an uncommon mechanism for myeloma kidney. Furthermore, the present case highlights an instance of morphologically and histologically rare findings of crystalline podocytopathy and tubulopathy in the kidney and crystalline deposition in plasma cells in the BM.

## Consent

Written informed consent was obtained from the patient for publication of this Case report and any accompanying images. A copy of the written consent is available for review by the Editor of this journal.

## Abbreviations

BM: Bone marrow
FSGS: Focal segmental glomerulosclerosis
Ig: Immunoglobulin
PCM: Plasma cell myeloma
